# Hyperhomocysteinemia is associated with decreased apolipoprotein AI levels in normal healthy people

**DOI:** 10.1186/s12872-016-0186-6

**Published:** 2016-01-13

**Authors:** Ying Wang, Jia Liu, Yuliang Jiang, Heng Zhang, Song Leng, Guang Wang

**Affiliations:** Physical Examination Center, Beijing Chao-yang Hospital, Capital Medical University, NO. 8, Gongti South Road, Chaoyang district, Beijing, 100020 China; Department of Endocrinology, Beijing Chao-yang Hospital, Capital Medical University, NO. 8, Gongti South Road, Chaoyang District, Beijing, 100020 China; Health Management Center, The Second Hospital of Dalian Medical University, NO. 467, Zhongshan Road, Shahekou District, Dalian, 116000 China

**Keywords:** Hyperhomocysteinemia, Apolipoprotein AI, Cardiovascular disease

## Abstract

**Background:**

Hyperhomocysteinemia (HHcy) is an independent risk factor for various cardiovascular diseases. Animal studies have shown that homocysteine (Hcy) inhibits hepatic expression of apolipoprotein AI (apoAI). Our recent clinical study showed that increased plasma Hcy levels were associated with decreased apoAI levels in patients with impaired glucose tolerance. In this study, we assessed a potential association between Hcy and apoAI levels in normal healthy people.

**Methods:**

A total of 1768 normal healthy individuals were divided into two groups: the control group (subjects without HHcy) and the HHcy group (subjects with HHcy).

**Results:**

HHcy subjects exhibited significantly lower high-density lipoprotein cholesterol (HDL-C) and apoAI levels than the control group (HDL-C: 1.18 ± 0.25 vs. 1.29 ± 0.32 mmol/L; apoAI: 1.38 ± 0.19 vs. 1.47 ± 0.25 g/L; all *P* < 0.01). Plasma Hcy levels were negatively associated with HDL-C and apoAI levels after adjustments for age, BMI and TG (HDL-C: *r* = –0.10; apoAI: *r* = –0.11; all *P* < 0.05). Multivariate regression analysis showed that the plasma Hcy levels were an independent influencing factor for apoAI (*β* = –0.065, *P* < 0.05).

**Conclusions:**

Increased plasma Hcy levels were associated with decreased apoAI levels in normal healthy people, and the inhibition of apoAI synthesis might be a mechanism through which Hcy is linked with the development of atherosclerosis in HHcy subjects.

## Background

Homocysteine (Hcy) is an intermediate of methionine metabolism [1]. Some genetic defects and nutritional deficiencies of B vitamins will cause elevated plasma Hcy levels, which is defined as hyperhomocysteinemia (HHcy) [1, 2]. HHcy has been widely proposed to be an independent risk factor for various cardiovascular diseases [3]. However, thus far, the precise mechanisms of how Hcy interferes with cardiovascular systems have not yet been revealed. In our previous studies, patients with acute coronary syndrome had increased plasma Hcy levels and HHcy subjects showed significant coronary endothelial dysfunction [4, 5]. Hcy increased the expression of monocyte chemoattractant protein-1 and promoted the generation of reactive oxygen species in human monocytes [6]. In white adipocytes, Hcy induced endoplasmic reticulum stress, unregulated the expression of resistin, and further caused insulin resistance [7, 8].

High-density lipoprotein (HDL) is a good lipoprotein with cardiovascular protective effects due to its anti-atherosclerotic, anti-inflammatory and anti-oxidative mechanisms [9]. ApoAI is a major component of HDL [10]. Our recent clinical study showed that increased plasma Hcy levels were associated with decreased apoAI levels in patients with impaired glucose tolerance (IGT) [11]. Previous research in male patients with coronary artery disease also demonstrated this negative correlation between Hcy and apoAI [12]. However, the associations between Hcy and apoAI levels in normal healthy people have not been well-characterized. In this study, we assessed the potential association between Hcy and apoAI levels in normal healthy people.

## Methods

### Study population

We enrolled 1768 normal healthy individuals over the age of 20 years who had undergone a routine physical examination at Beijing Chao-yang Hospital Affiliated to Capital Medical University from March 2012 to October 2014. Oral glucose tolerance tests (OGTT) and blood pressure measurements were performed at screening. Individuals with hypertension, coronary artery disease, diabetes, pre-diabetes, liver or renal function impairment, infectious disease, systemic inflammatory disease or cancer were excluded. No subject took lipid-lowering agents or vitamins. HHcy was defined by the plasma Hcy level >15 μmol/L [2]. All participants were divided into two groups: the control group (subjects without HHcy) and HHcy group (subjects with HHcy). All enrolled subjects provided written informed consent. The protocol of this study was approved by the Ethics Committee of the Beijing Chao-yang Hospital Affiliated to Capital Medical University.

### Clinical and biochemical measurements

A standard questionnaire was used to collect information about the patients’ health status and medications. Height and weight were measured to the nearest 0.1 cm and 0.1 kg, respectively, by the same trained group. Blood pressure was measured from the non-dominant arm in a seated position after a ten-minute rest using a sphygmomanometer. Venous blood samples were obtained after overnight fasting. Plasma samples of all participants were stored at –80 °C. High-density lipoprotein cholesterol (HDL-C), low-density lipoprotein cholesterol (LDL-C), triglyceride (TG), total cholesterol (TC) levels were measured by colorimetric enzymatic assays using an autoanalyzer (Hitachi 7170). Plasma TC was measured by an enzymatic cholesterol oxidase reaction, TG by a glycerol lipase oxidase reaction, and HDL-C and LDL-C were measured by the direct assay. ApoAI and apolipoprotein B (apoB) were analyzed with immune turbidimetry. Hcy, fasting blood glucose (FBG), and fasting insulin (FINS) levels were measured at the central chemistry laboratory in Beijing Chao-yang Hospital Affiliated to Capital Medical University. Plasma Hcy levels were measured by the clinical chemistry, FBG by the glucose oxidase method, and FINS were measured by the chemiluminescence method. Body mass index (BMI) was calculated as the weight in kilograms divided by the height in meters squared. Homeostasis model assessment of insulin resistance (HOMA-IR) was performed to evaluate insulin resistance according to the following formula: HOMA-IR = [FINS (μIU/mL) * FBG (mmol/L)/22.5] [13].

### Statistical methods

Normally distributed variables were expressed as the mean ± standard deviation (SD), while variables with a skewed distribution, including TG, FINS and HOMA-IR, were given as the median and upper and lower quartiles. Variables that were not normally distributed were log-transformed before analysis. The proportions were analyzed using chi-square tests. We also performed Pearson and Spearman correlation analyses. Multivariate analysis was used to evaluate the correlation. All statistical analyses were performed with SPSS 17.0 (SPSS, Inc., Chicago, IL), and the results were considered statistically significant with two-tailed analyses, *P* < 0.05.

## Results

### Clinical characteristics of the control and HHcy groups

The clinical characteristics of participants were summarized in Table 1. The control and HHcy groups did not significantly differ in age, gender, BMI, SBP and DBP. The levels of FBG, FINS and HOMA-IR were also statistically the same between the two groups. HHcy subjects showed higher TG and lower HDL-C and apoAI levels compared to controls [TG: 1.28 (0.88–1.97) vs. 1.12 (0.76–1.71) mmol/L; HDL-C: 1.18 ± 0.25 vs. 1.29 ± 0.32 mmol/L; apoAI: 1.38 ± 0.19 vs. 1.47 ± 0.25 g/L; all *P* < 0.01]. However, there was no significant difference in the TC, LDL-C and ApoB levels between the two groups.

**Table 1:** Baseline characteristics of the control and HHcy groups

Parameters	Control group (*n* = 1412)	HHcy group (*n* = 356)
Age, y	41.11 ± 11.44	41.25 ± 12.25
Gender, Males/Females	790/622	196/160
BMI, kg/m^2^	24.33 ± 3.44	24.68 ± 3.13
SBP, mmHg	120.34 ± 11.26	121.35 ± 11.19
DBP, mmHg	73.04 ± 8.65	73.72 ± 8.89
TC, mmol/L	4.89 ± 0.90	4.87 ± 0.85
LDL-C, mmol/L	2.83 ± 0.73	2.90 ± 0.71
HDL-C, mmol/L	1.29 ± 0.32	1.18 ± 0.25**
TG, mmol/L	1.12 (0.76–1.71)	1.28 (0.88–1.97)**
apoAI, g/L	1.47 ± 0.25	1.38 ± 0.19**
apoB, g/L	0.87 ± 0.20	0.90 ± 0.20
FBG, mmol/L	5.34 ± 0.36	5.38 ± 0.35
FINS, μIU/mL	11.25 (7.40–16.16)	11.41 (8.39–16.40)
HOMA-IR	2.68 (1.74–3.87)	2.72 (1.97–4.01)
Homocysteine, μmol/L	11.04 ± 1.81	26.53 ± 13.68**

Data are means ± SD unless indicated otherwise. TG, FINS and HOMA-IR are shown as median and range. HHcy: hyperhomocysteinemia; BMI: body mass index; SBP: systolic blood pressure; DBP: diastolic blood pressure; FBG: fasting blood glucose; TC: total cholesterol; LDL-C: low-density lipoprotein cholesterol; HDL-C: High-density lipoprotein cholesterol; TG: triglyceride; apoAI: apolipoprotein AI; apoB: apolipoprotein B; FINS: fasting insulin; HOMA-IR: homeostasis model assessment of insulin resistance. * significantly different at *P* < 0.05 vs control; ** significantly different at *P* < 0.01 vs control

### Correlation between plasma Hcy and the levels of apoAI or other lipid profiles

**Fig. 1:**
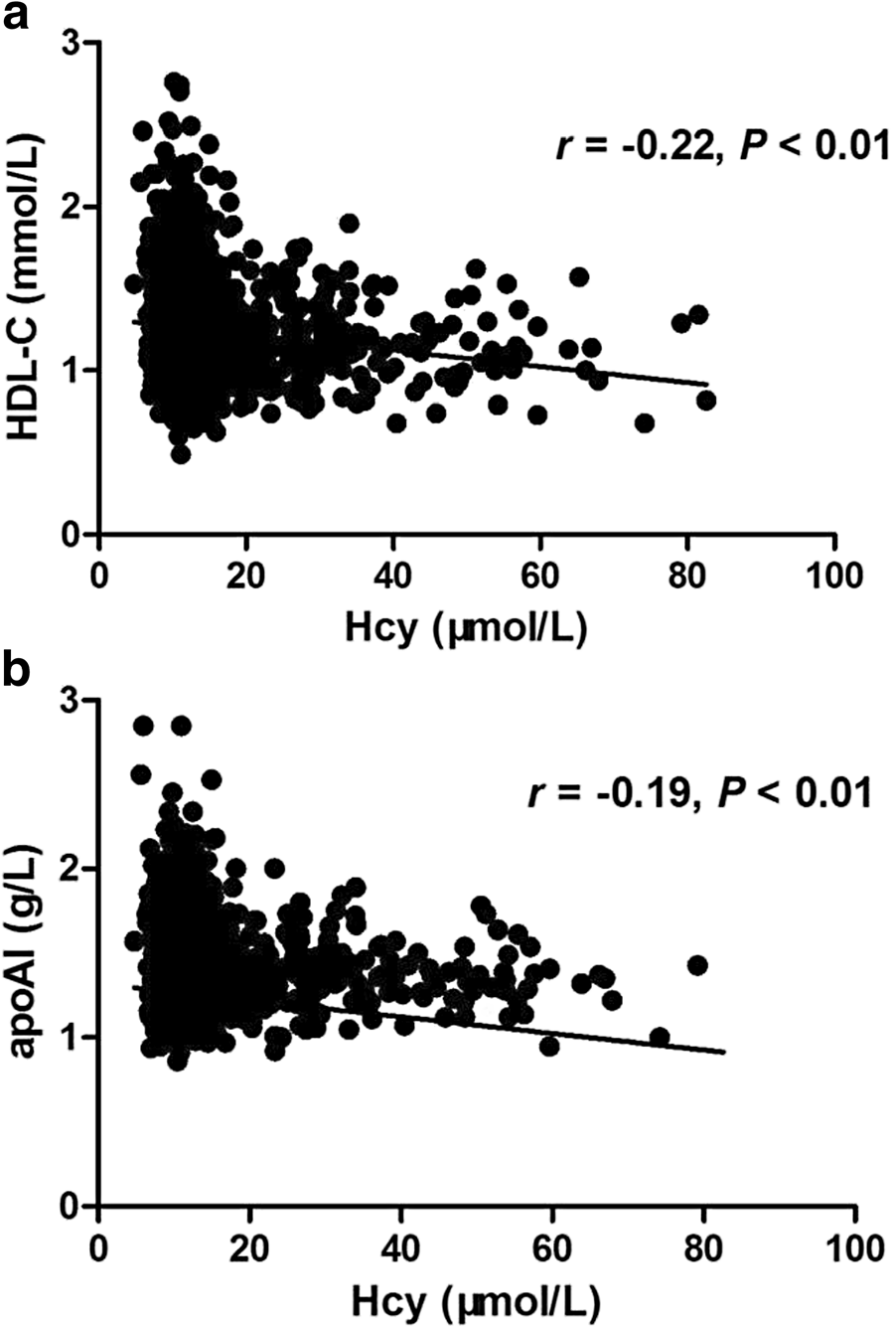
The correlation between plasma Hcy and the levels of HDL-C (**a**) and apoAI (**b**). Hcy were negatively correlated with the levels of HDL-C and apoAI (HDL-C: *r* = –0.22, *P* < 0.01, 95 % confidence interval –0.27 to –0.16) (apoAI: *r* = –0.19, *P* < 0.01, 95 % confidence interval –0.24 to –0.13), and this negative correlation was still observed after adjustments for age and BMI levels

Hcy was negatively correlated with the levels of HDL-C and apoAI (HDL-C: *r* = –0.22, *P* < 0.01, 95 % confidence interval –0.27 to –0.16; Fig. 1a) (apoAI: *r* = –0.19, *P* < 0.01, 95 % confidence interval –0.24 to –0.13; Fig. 1b), and this negative correlation was observed after adjusting for age, BMI and TG levels (HDL-C: *r* = –0.10; apoAI: *r* = –0.11; all *P* < 0.05). We also found a positive correlation between Hcy and the levels of TG and LDL-C (TG: *r* = 0.14, *P* < 0.01, 95 % confidence interval 0.08 to 0.19) (LDL-C: *r* = 0.07, *P* < 0.01, 95 % confidence interval 0.02 to 0.12); however, these correlations disappeared after adjustments for age and BMI.

### Correlation between plasma Hcy and other metabolic parameters

In all participants, we did not find a correlation between Hcy and other metabolic parameters, including age, BMI, SBP, DBP, FBG, FINS and HOMA-IR. However, Hcy was positively correlated with the levels of SBP and DBP in the HHcy group (SBP: *r* = 0.12, *P* < 0.01, 95 % confidence interval 0.07 to 0.17) (DBP: *r* = 0.14, *P* < 0.01, 95 % confidence interval 0. 08 to 0.19), and these correlations remained even after adjusting for age and BMI levels (SBP: *r* = 0.09; DBP: *r* = 0.10; all *P* < 0.05).

### Multivariate stepwise regression analysis

To further evaluate the relationship between plasma homocysteine and apoAI levels, we performed a multivariate regression analysis, including age, gender, BMI, TG, Hcy and HOMA-IR. We found that plasma Hcy levels were an independent influencing factor for apoAI levels (*β* = –0.065, *P* < 0.05).

## Discussion

In the present study, we assessed the potential association between Hcy and apoAI levels in normal healthy people. HHcy subjects exhibited significantly lower HDL-C and apoAI levels than the control group. The plasma Hcy levels were negatively associated with HDL-C and apoAI levels after adjusting for age, BMI and TG. Multivariate regression analysis showed that the plasma Hcy level was an independent influencing factor for apoAI.

HHcy is often associated with essential hypertension [14]. Our previous studies showed that IGT patients with HHcy had a higher prevalence of essential hypertension [11]. HHcy increased blood pressure mediated through the disruption of the bioavailability of tetrahydrobiopterin and caused vascular endothelial dysfunction [14]. In the present study, we observed healthy participants without essential hypertension and found that Hcy was positively correlated with the SBP and DBP levels in the HHcy group. Subjects with a high-normal blood pressure exhibited decreased artery elastic function and endothelial dysfunction [15, 16]. Thus, HHcy may potentially enhance the levels of blood pressure even within the normal range, and elevated blood pressure is one factor that is likely to mediate cardiovascular disease in HHcy patients.

Our previous studies showed that Hcy contributes to insulin resistance by inducing endoplasmic reticulum stress, upregulating the expression of resistin, and interrupting the phosphorylation of insulin receptor tyrosine kinase [17]. In IGT patients, plasma Hcy levels are positively related to HOMA-IR [11]. Hypertensive patients with HHcy had significant insulin resistance compared to patients without HHcy [5]. However, in the present study, there was no correlation between Hcy and HOMA-IR not only in all of the participants but also in the HHcy subjects. Insulin resistance is associated with obesity, dyslipidemia and hypertension and other metabolic risk factors [18]. IGT and hypertensive patients had obvious metabolic disorders [19]; however, the present study investigated normal healthy people who did not have hypertension, diabetes and other overt metabolic diseases. Thus, it might suggest that HHcy had a synergistic promoting effect on insulin resistance with metabolic risk factors, but HHcy had a weaker effect by itself on insulin resistance compared to hypertension, diabetes and obesity.

HDL is a lipoprotein that is produced in the liver [9]. It has anti-atherogenic properties by transporting cholesterol from cells into peripheral tissues, reducing oxidative stress and suppressing inflammatory pathways [9]. ApoAI is a major functional component of HDL and is extensively involved in the cardiovascular protective effects of HDL [10]. Transgenic over-expression of apoAI mice exhibit elevated plasma HDL-C levels and reduced vascular lesions [20]. Low apoAI (HDL-C) levels are a risk factor for atherosclerosis [9, 21]. However, the underlying mechanism of low apoAI (HDL-C) levels still remains unknown. Some factors, including obesity and smoking, have been reported to reduce HDL-C and apoAI levels [22]. Recent studies have presented a correlation between Hcy and apoAI. Recent animal and in vitro cell studies have also demonstrated that Hcy suppresses hepatic apoAI expression via the peroxisome proliferator activated receptor α (PPARα) - apoAI pathway [12, 23, 24]. Moreover, Hcy could decrease the transcription of apoAI by stimulating nuclear factor κB (NF-κB) and apoAI regulatory protein-1 (ARP-1) [19, 25]. Decreased plasma and hepatic expression levels of apoAI have also been observed in animal models of HHcy [12, 26]. Data from population studies in IGT patients and male patients with coronary artery disease showed that Hcy levels are negatively correlated with apoAI levels [12]. In the present study, plasma Hcy levels were negatively associated with the HDL-C and apoAI levels in normal healthy people, and plasma Hcy levels were an independent influencing factor for apoAI levels. Thus, it might suggest that HHcy is involved in the development of low plasma levels of apoAI and HDL-C in healthy people, and the inhibition of apoAI synthesis is a subsequent mechanism through which Hcy is linked to atherosclerosis development in HHcy patients. However, inconsistently, a recent study showed that the plasma folate, but not Hcy, is associated with apoAI levels in a non-fortified population [27]. One possible explanation for this opposing result is that not all participants in that study were fasting at the time of blood sampling, and the levels of Hcy, folate and blood lipid may be influenced by eating [27]. Unfortunately, our study did not assess the serum folate levels of the participants. We will address this issue in our further research.

Our study has several limitations. The major limitation was that we did not assess the serum folates and MTHFR polymorphisms (677C > T and 1298A > C) which had been demonstrated as the determinant of the plasma Hcy levels. Additionally, as a cross-sectional study, it is hard to confirm causality. Further prospective or intervention researches are needed to evaluate the potential association between Hcy and apoAI levels.

## Conclusions

In conclusion, increased plasma Hcy levels were associated with decreased apoAI levels in normal healthy people, and the inhibition of apoAI synthesis might be a mechanism through which Hcy is linked with the development of atherosclerosis in HHcy subjects. Further researches are needed to evaluate the potential association between Hcy and apoAI levels.
